# Design and Synthesis of a Novel ICT Bichromophoric pH Sensing System Based on 1,8-Naphthalimide Fluorophores as a Two-Input Logic Gate and Its Antibacterial Evaluation

**DOI:** 10.3390/molecules28083631

**Published:** 2023-04-21

**Authors:** Alaa R. Sakr, Nikolai I. Georgiev, Vladimir B. Bojinov

**Affiliations:** 1 Department of Organic Synthesis, University of Chemical Technology and Metallurgy, 8 Kliment Ohridsky Str., 1756 Sofia, Bulgaria; alaasakr81@yahoo.com (A.R.S.); nikigeorgiev@uctm.edu (N.I.G.); 2 Department of Chemistry, Faculty of Science, Zagazig University, Zagazig 44519, Egypt; 3 Bulgarian Academy of Sciences, 1040 Sofia, Bulgaria

**Keywords:** 1,8-naphthalimide, ICT bichromophore, pH sensing, strip paper indicator, INHIBIT and IMPLICATION logic gates, fluorescence, antibacterial activity

## Abstract

The synthesis, sensor activity, and logic behavior of a novel 4-iminoamido-1,8-naphthalimide bichromophoric system based on a “*fluorophore-receptor*” architecture with ICT chemosensing properties is reported. The synthesized compound showed good colorimetric and fluorescence signaling properties as a function of pH and proved itself as a promising probe for the rapid detection of pH in an aqueous solution and base vapors in a solid state. The novel dyad is able to work as a two-input logic gate with chemical inputs H^+^ (Input 1) and HO^−^ (Input 2) executing INHIBIT logic gate. The synthesized bichromophoric system and the corresponding intermediates demonstrated good antibacterial activity toward Gram (+) and Gram (−) bacteria when compared with the Gentamycin standard.

## 1. Introduction

Chemical sensor technology can provide low-cost devices that can be tuned to a wide field of applications by coating mass-sensitive or optical transducers with a chemically sensitive layer [1]. Fluorescent devices for sensing and reporting chemically and biologically important species are currently of great importance to chemistry, biology, and environmental science [2]. Fluorescent probes have received much attention due to their advantages such as high sensitivity, high selectivity, fast response time, and inexpensive instrumentation [3,4,5]. They are also excellent diagnostic tools in medicine and biology, as they allow the monitoring of events in living cells and organisms [6,7,8,9]. Due to their extremely small size, the fluorescent probes can penetrate and work in living organisms without threatening their life [10,11,12].

Two common principles are used for the construction of fluorescent probes: photoinduced electron transfer (PET) and intramolecular charge transfer (ICT) [13,14,15]. Unlike PET-based fluorescent molecular structures, where the receptor is separated from the fluorophore by a spacer [16,17,18,19], in ICT sensory systems, the receptor is directly attached to the conjugated system of the fluorophore [20,21,22]. Upon excitation, an environmentally dependent intramolecular charge transfer occurs in the fluorophore-conjugated system. Thus, recognition of the guest affects ICT efficiency, which changes the energy between the ground and excited state and results in shifting of the fluorophore electronic spectra [23,24,25].

Heterocyclic 1,8-naphthalimide derivatives are ICT fluorophores with remarkable optoelectronic characteristics. Light absorption in these molecules is known to generate a strongly environment-dependent charge transfer interaction between the C-4 substituent and the carbonyl groups. Thanks to their excellent properties, the 1,8-naphthalimides are used in a variety of applications [26,27].

A significant goal in the area of nanotechnology is the design of molecular devices with built-in individually functional components that work cooperatively as a whole [28]. Supramolecular devices exhibiting significant differences in their “*off*” and “*on*” states are currently the subject of considerable interest, as they can be modulated or tuned using external sources such as ions, molecules, light, etc. [29,30,31,32,33,34]. The “*off*” and “*on*” states of the molecular-level devices refer to their luminescence, magnetic, or electronic properties [35,36,37,38,39,40,41,42]. The binary options, in which the signal change is large enough to be considered “*off–on*” or “*on–off*” are increasingly conscious of information technology. A. de Silva later demonstrated A. Aviram’s theoretical concept of molecular logic [43] experimentally by establishing an analogy between molecular switches and electronic logic gates [44]. As a result, a large number of the most important logic functions have been implemented at the molecular level to date [45,46,47,48,49]. In particular, the photophysical phenomena of the fluorescence architectures occurring in the excited state (proton, energy, electron, or charge transfer) have very often been used for the rational design of molecular logic devices [50,51]. These molecules, capable of performing various sensing functions and computing a composite result autonomously, have great potential for real-life applications, such as smart materials, drug delivery and activation, object and image encoding, diagnostics, or actuation [52,53,54,55]. Over almost two decades, practically all of the fundamentally important logic gates [56,57,58,59,60,61,62,63] and systems such as half adder/subtractor [64,65], full adder/subtractor [66], multiplexer [67], encoder/decoder [68], digital comparator [69,70,71,72], and keyboard lock [73] were achieved.

**Scheme 1:**
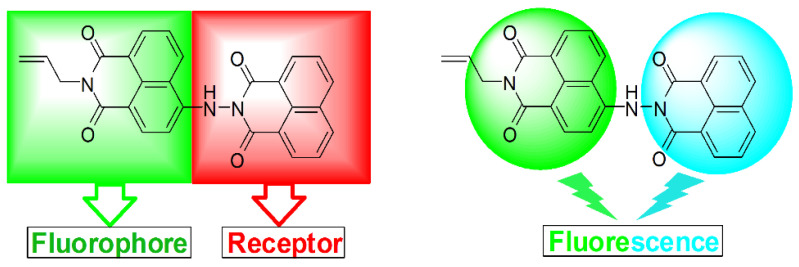
Design of Dyad **5** on the “*fluorophore-receptor*” model.

Herein, we report on the design, synthesis, and sensor activity of a novel 4-iminoamido-1,8-naphthalimide ICT fluorescent probe (Scheme 1). The fluorescence chemosensing behavior of the synthesized compound was studied, and the results obtained clearly demonstrated the high potential of the novel probe to determine pH in aqueous solutions and solid states. pH is one of the most important chemical parameters that plays a key role in chemical laboratories, clinical analysis, food production, biotechnological processes, wastewater treatment, and the environmental and life sciences [74,75,76,77,78]. Furthermore, intracellular and extracellular pH is a crucial physiological parameter for which measurement is very useful for cellular analysis or medical diagnostics [79,80,81,82,83,84]. The logic behavior and antibacterial activity of the novel compound were also investigated.

## 2. Results and Discussion

### 2.1. Design and Synthesis of Bichromophoric System ***5***

The 4-iminoamido-1,8-naphthalimide **5** represents a “*fluorophore-receptor*” architecture with ICT chemosensing properties, in which 4-imino-1,8-naphthalimide is the fluorophore and the iminoamido group, possessing a labile proton, is the receptor fragment. The fragments with labile N-H bonds are widely used for anion detection because the acidity of the NH group can be easily tuned by adjusting the electronic properties of neighboring substituents so that it can recognize anions in the surrounding environment either by hydrogen bonding or by deprotonation of the labile hydrogen atom.

It is well known that the absorption and fluorescence characteristics of the 1,8-naphthalimides depend on the nature of the substituent at a C-4 position of the 1,8-naphthalimide ring. The 4-imino-1,8-naphthalimide is a “*push–pull*” π-electron system in which the absorbed light induces charge transfer from the donor amine at the C-4 position of the 1,8-naphthalimide to the two peripositioned carbonyl acceptors. When an anion, such as OH^−^, interacts with the imino group in the 4-imino-1,8-naphthalimide fluorophore, the electron-donating ability of the imino group increases due to its deprotonation, which generates a strong electron density around the imino nitrogen. As a result, the efficiency of intramolecular charge transfer in the 1,8-naphthalimide fluorophore increases sharply and leads to a shift in the absorption maximum to longer wavelengths.

**Scheme 2:**
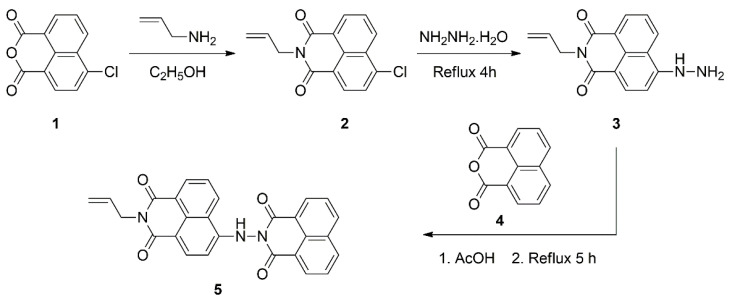
Synthesis of probe **5**.

The synthesis of the novel 1,8-naphthalimide **5** was performed in three steps, as it is shown in Scheme 2. First, the intermediate 4-chloro-*N*-allyl-1,8-naphthaimide **2** was obtained by the reaction of 4-chloro-1,8-naphthalic anhydride **1** with allylamine. Then the chlorine atom in **2** was nucleophilically substituted with hydrazine to give 4-hydrazinyl-*N*-allyl-1,8-naphthalimide **3**. The final Dyad **5** was obtained by the reaction of intermediate **3** with 1,8-naphthalic anhydride. The reaction was performed under reflux in glacial acetic acid. The pure product was isolated by filtration of a precipitate that had formed on cooling the reaction mixture.

The photophysical properties of 4-substituted-1,8-naphthalimides are directly dependent on the polarization of the molecule and may be influenced by the environmental effect of the media. As can be seen from Table 1, in water/DMF (3:1, *v*/*v*) solution, compound **2**, which contains a chlorine atom in the C-4 position, absorbs in the UV region at *λ*_A_ = 342 nm. The replacement of the chlorine atom (electron-acceptor) with a hydrazide (electron-donor) group bathochromically shifts the absorption of compound **3** in the visible region of the spectrum at *λ*_A_ = 448 nm.

The synthesized compounds were characterized and identified by melting points, *R*_f_ values, elemental analysis, and ^1^H and ^13^C NMR spectra (Figures S3–S10).

The photophysical properties of probe **5** were also studied in solvents of different polarities (Table S1). A typical bathochromic shift in the absorption and emission bands was observed with increasing solvent polarity. No specific effects were observed in protic ethanol, indicating a lack of intermolecular H-bond formation in the ground state.

**Figure 1:**
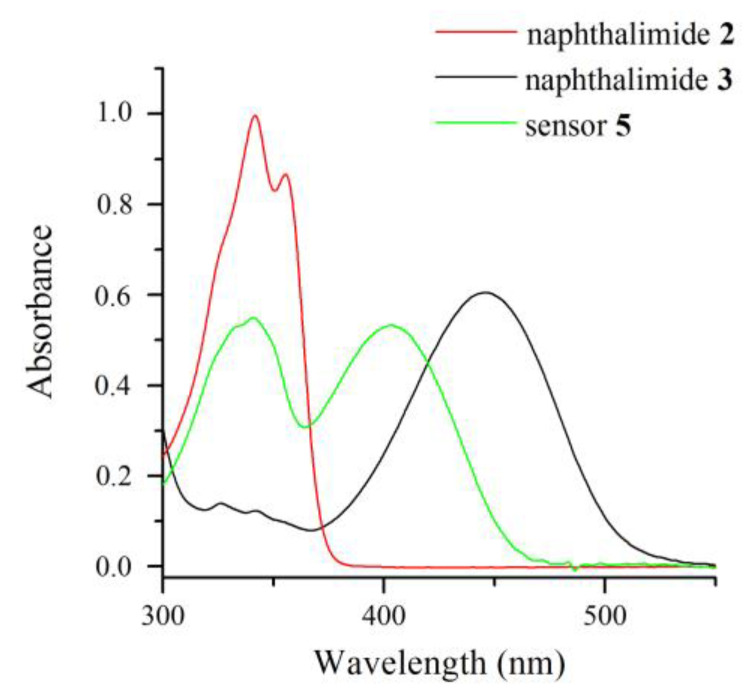
Absorption spectra of 1,8-naphthalimides **2** and **3** and probe **5**.

Due to the reduced electron-donating ability of the NH group in the 4-imino-1,8-naphthalimide sensor **5**, the absorption maximum in the visible region was moved hypsochromically from 448 nm for compound **3** to 404 nm for compound **5** (Figure 1).

As can be expected, compound **5** possesses two absorption bands corresponding to the absorptions of the individual chromophores. From Figure 1, where the normalized spectra of compounds **2**, **3**, and **5** are presented, it is clearly seen that the spectrum of sensor **5** is a sum of unsubstituted (*λ*_A_ = 342 nm) and 4-imino substituted naphthalimide (*λ*_A_ = 404 nm).

### 2.2. Influence of pH on the Absorption and Fluorescence Characteristics of Bichromophoric System ***5***

The compound **5** under study was designed as a fluorescence sensor for the determination of pH changes over a wide pH scale. This was the reason for investigating the photophysical behavior of compound **5** in water/DMF (3:1, *v*/*v*) solution at different pH values. The investigation was conducted in water/DMF solution to prevent the aggregation of compound **5** in pure water.

When 1,8-naphthalimide fluorophores contain an NH-acidic receptor fragment, one would expect that in the presence of anions, the receptor fragment would be deprotonated, resulting in a sharp increase and bathochromic shift in the absorption of 4-imino-1,8-naphthalimide.

**Figure 2:**
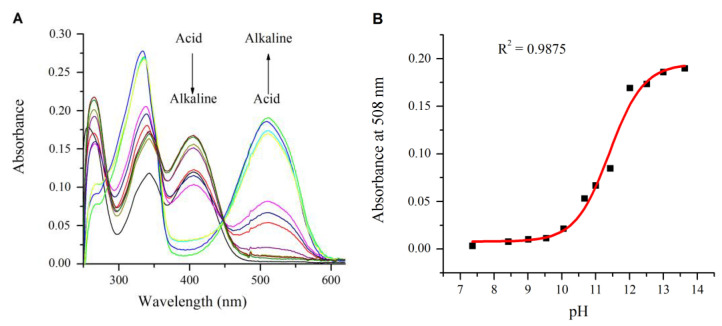
(**A**) Absorption of sensor **5** at different pH and (**B**) titration plot of **5** at *λ*_A_ = 508 nm in a pH range of 7–14.

**Figure 3:**
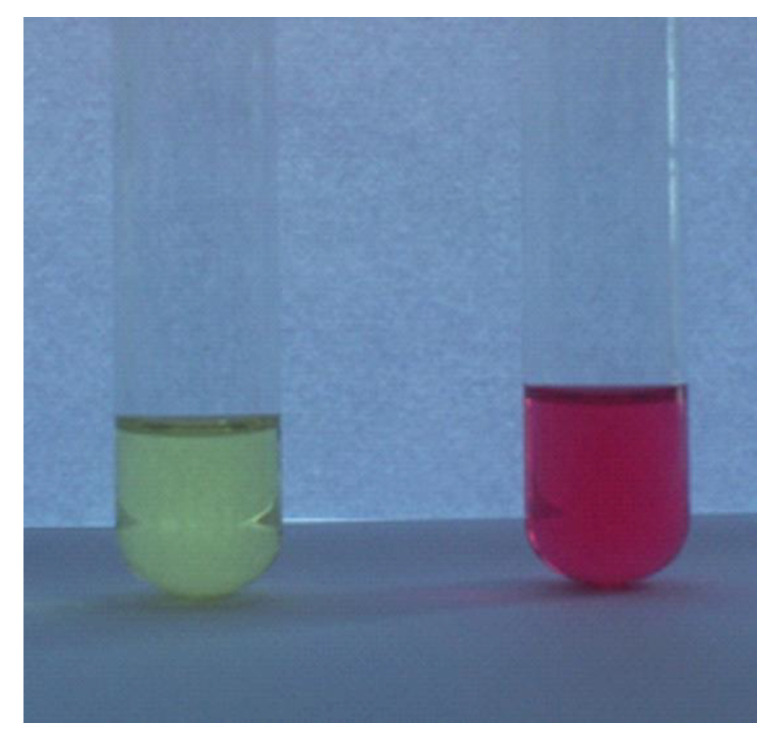
Colorimetric changes of **5** in the absence (**left**) and in the presence of NaOH (**right**).

Taking into account the above assumption, the photophysical behavior of compound **5** was investigated in the presence of hydroxyl ions (NaOH). The solution of **5** was colored yellow-green and showed two absorption bands at 342 nm and 404 nm (Figure 2A). As expected, the addition of hydroxyl ions to the system led to the appearance of a new absorption band at 508 nm, which changed the color of the probe to red (Figure 3). At the same time, the absorption band at 404 nm disappeared completely (Figure 2A).

In a pH range ca. 2–7, as expected, Dyad **5** retained a weak absorbance at 508 nm due to an inability to deprotonate the imide nitrogen (Figure S1).

From the absorbance changes at 508 nm as a function of pH in a pH range of 714, a well-pronounced S-shaped (sigmoidal Boltzmann fit, R^2^ = 0.9875) titration plot was obtained (Figure 2B). The analysis of the absorbance changes according to Henderson–Hasselbalch Equation (1) [85] in a pH window 7–14 (Figure 2B) gives a p*K*_a_ value of 11.40 ± 0.089 for the deprotonated form of Dyad **5**.

(1) $$pH=pK_{a}+\log\frac{\left(A-A_{max}\right)}{\left(A-A_{min}\right)}$$

where *A_max_* and *A_min_* are the maximum and minimum absorbances, respectively, and *A* is the absorbance at the corresponding pH value.

**Figure 4:**
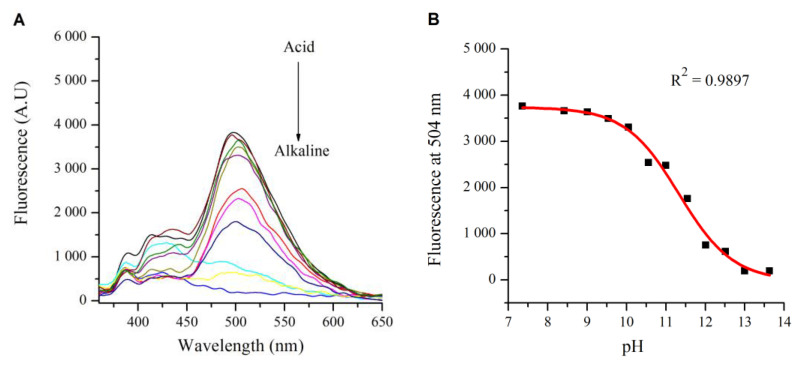
(**A**) Fluorescence of sensor **5** at different pH (*λ*_EX_ = 340 nm) and (**B**) titration plot of **5** at *λ*_F_ = 504 nm in a pH range of 7–14 (*λ*_EX_ = 340 nm).

After excitation at 340 nm in the absorption maximum of the unsubstituted at C-4 position 1,8-naphthalimide part of the molecule, the system showed two emission bands at about 410 nm and at 504 nm, which was quenched upon transition from an acid to an alkaline environment (Figure 4A).

**Scheme 3:**
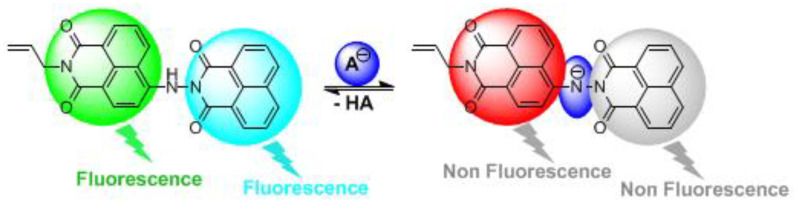
Photophysical behavior of compound **5** in the presence of anions.

The two bands at about 410 nm (weak fluorescent intensity) and at 504 nm (strong fluorescent intensity) are the result of energy transfer from the donor to the acceptor fluorophore, which is possible because the emission maximum of the unsubstituted at C-4 position 1,8-naphthalimide overlaps very well with the absorption maximum of 4-imino-1,8-naphthalimide. Consequently, the acceptor fluorophore is excited and emits at 504 nm. Upon transition to an alkaline medium, after deprotonation of the imino group, the absorption maximum of the acceptor fluorophore is significantly red-shifted, due to which the energy transfer becomes impossible, and the emission at 504 nm is quenched (Scheme 3).

From the fluorescence changes at 504 nm (*λ*_EX_ = 340 nm) as a function of pH in a pH window 7–14, a well-pronounced S-shaped (sigmoidal Boltzmann fit, R^2^ = 0.9897) titration plot was observed for the deprotonation of compound **5** (Figure 4B).

According to Henderson–Hasselbalch Equation (2) [86], a very similar p*K*_a_ value (p*K*_a_ = 11.33 ± 0.12) was calculated from the fluorescence changes at 504 nm (*λ*_EX_ = 340 nm) as a function of pH in a window of 7–14 (Figure 4B), which suggests a simple equilibrium by the deprotonation of Dyad **5**.

(2) $$pH=pK_{a}+\log\frac{\left(I-I_{max}\right)}{\left(I-I_{min}\right)}$$

where *I_max_* and *I_min_* are the maximum and minimum fluorescence intensities, respectively, and *I* is the fluorescence intensity at the corresponding pH value.

**Figure 5:**
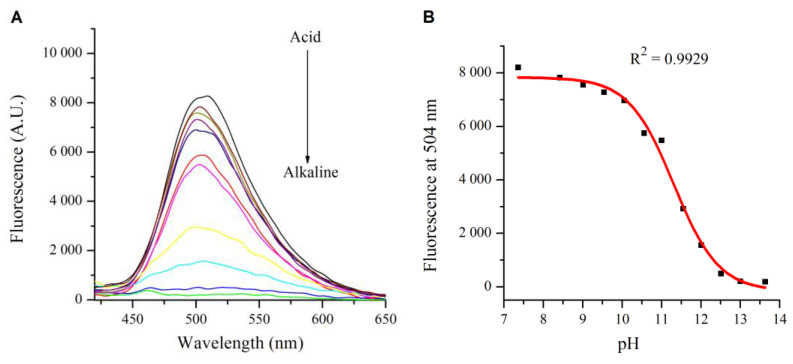
(**A**) Fluorescence of sensor **5** at different pH (*λ*_EX_ = 400 nm) and (**B**) titration plot of **5** at *λ*_F_ = 504 nm in a pH range of 7–14 (*λ*_EX_ = 400 nm).

After direct excitation of the 4-imino-1,8-naphthalimide part of the molecule at 400 nm, the emission band at 504 nm disappeared in alkaline media as well due to deprotonation of the aromatic NH group (Figure 5A). From the fluorescence changes at 504 nm (*λ*_EX_ = 400 nm) as a function of pH in a pH window 7–14, a well-pronounced S-shaped (sigmoidal Boltzmann fit, R^2^ = 0.9929) titration plot was obtained for the deprotonation of compound **5** (Figure 5B).

In a pH window ca. 2–7, due to the impossibility of deprotonation of the aromatic NH group, Dyad **5** retained its high levels of fluorescence intensity practically without any change up to about pH = 8 (Figure S2).

According to Henderson–Hasselbalch Equation (2) [86], again, a very similar p*K*_a_ value (p*K*_a_ = 11.31 ± 0.08) was calculated from the fluorescence changes at 504 nm (*λ*_EX_ = 400 nm) as a function of pH in a window of 7–14 (Figure 5B), which suggests, once again, a simple equilibrium by the deprotonation of Dyad **5**.

It can be summarized that upon addition of hydroxyl ions to a solution of Dyad **5** in water/DMF (3:1, *v*/*v*) at the transition from acidic to alkaline conditions, the imino group of the probe is deprotonated, which induces a process of gradual decreasing of its fluorescence intensity with increasing pH of the medium.

For the decrease in fluorescence intensity of probe **5**, excited at 340 nm, a qualitative parameter fluorescence quenching FQ = 30.1 was obtained. The FQ = *I*/*I*_0_ is the ratio between the maximum fluorescence intensity *I_0_* at ca. pH 7 and the minimum fluorescence intensity *I* at ca. pH 14 (Figure 4B). For the fluorescence quenching of probe **5** after excitation at 400 nm, a value of FQ = 68.5 was calculated (Figure 5B).

**Figure 6:**
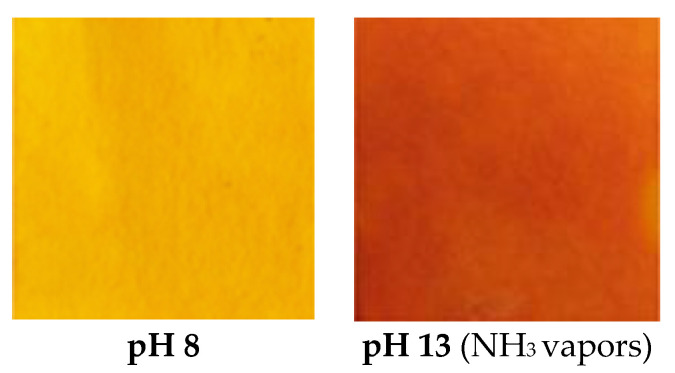
Fluorescent response of strip papers based on 1,8-naphthalimide **7** at different pHs.

Due to portability, very low cost, and easier operation, paper is a useful material for the fabrication of chemosensing devices [87,88,89,90,91]. That is why the extension of the principles of molecular sensors from liquid solution onto solid support could open up new directions for practical applications [92,93,94,95]. Motivated by this principle, in order to obtain strip papers with chemosensing properties based on **5**, a methanolic solution of **5** was poured onto a filter paper, and the solvent was evaporated. The prepared strip papers showed a highly sensitive colorimetric change from yellow to red toward basic vapors such as NH_3_. As can be seen from Figure 6, the exposure of **5-**based strip papers on NH_3_ vapors for 2 s resulted in a color change, which was detectable, even with the naked eye.

This result reveals the great ability of the **5-**based strip papers to serve as a promising chemosensing material for base vapors. Unfortunately, the red-colored strip paper was unstable, and the papers were returned back to yellow form after 2 h, which disallowed its application for the detection of acid vapors after treatment with base.

The performed study showed the high potential of the novel compound **5** as an efficient platform for the rapid detection of anions in aqueous solutions and base vapors in solid states.

### 2.3. Logic Gates of Probe ***5***

Based on its absorption behavior as a function of pH, Dyad **5** is able to operate as a two-input logic gate with chemical input protons (Input 1) and hydroxyl anions (Input 2).

**Figure 7:**
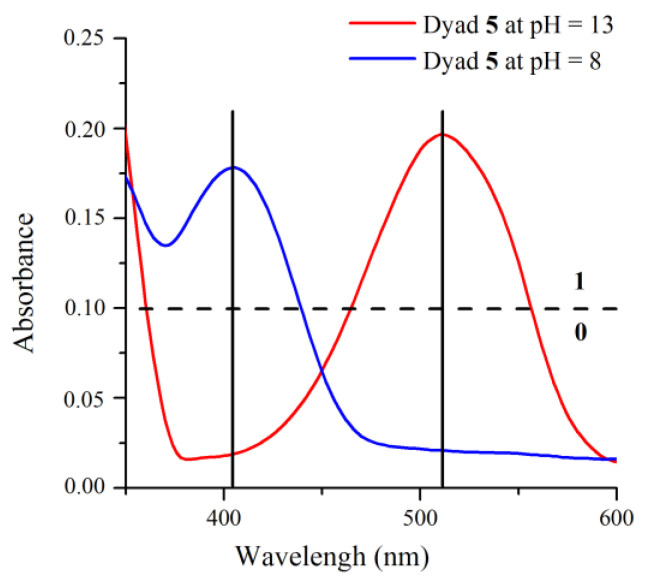
The changes in the absorbance of Dyad **5** at 404 nm (Output 1) and at 508 nm (Output 2) with two chemical inputs (Table 2).

First, using the absorption at 404 nm (Output 1, Table 2), an INHIBIT gate at the molecular level can be achieved. In starting alkaline solution (pH = 13), Dyad **5** is in deprotonated form with a low absorption band at 404 nm (coded for binary 0). After the addition of protons (Input 1), Dyad **5** is converted to its neutral form (pH = 8) with a yellow-green color and high absorption at 404 nm (coded for binary 1). The simultaneous inputs of acid and base annihilated each other, and Dyad **5** remains in a deprotonated form in which the absorption at 404 nm is low (coded for binary 0). Thus, the absorption at 404 nm of Dyad **5** is high only in the presence of H^+^ (Input 1) and in the absence of OH^−^ (Input 2), which is correlated very well with the INHIBIT gate. Due to the inverse connection between the absorptions at 404 nm and 508 nm (when the absorption at 404 nm is high, the absorption at 508 nm is low, and when the absorption at 404 nm is low, the absorption at 508 nm is high), Dyad **5** is able to execute the IMPLICATION gate, which is related inversely (negative logic) to the above INHIBIT gate. As can be seen, the absorption output of Dyad **5** at 508 nm (Output 2, Table 2) is low only in the presence of protons and in the absence of OH^−^. In all other cases, the output is high. This behavior mimics the IMPLICATION gate (Figure 7).

**Figure 8:**
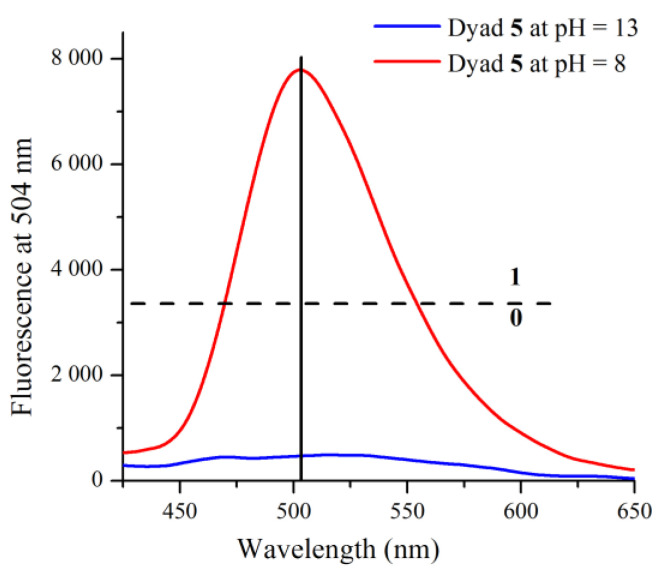
The changes in the fluorescence intensity of Dyad **5** at 504 nm (*λ*_EX_ = 400 nm) with two chemical inputs (Output 3, Table 2).

Monitoring the fluorescent intensity at 504 nm (*λ*_EX_ = 400 nm) as a function of pH (Output 3, Table 2), it can be easily seen that Dyad **5** also works successfully as a two-input logic gate with chemical input protons (Input 1) and hydroxyl anions (Input 2), and to execute the INHIBIT logic gate (Figure 8).

In starting alkaline solution (pH = 13), Dyad **5** is in deprotonated form with low fluorescence intensity at 504 nm (coded for binary 0). After the addition of protons (Input 1), Dyad **5** is transformed into its neutral form (pH = 8) with high fluorescent intensity at 504 nm (coded for binary 1). The simultaneous inputs of acid and base annihilate each other, and Dyad **5** remains in a deprotonated form in which the fluorescent intensity at 504 nm is low (coded for binary 0). Thus, the fluorescent intensity at 504 nm of Dyad **5** is high only in the presence of H^+^ (Input 1) and in the absence of OH^−^ (Input 2), which is correlated very well with the INHIBIT gate.

### 2.4. Antibacterial Evaluation of the Synthesized Dyes

The synthesized compounds were tested for antimicrobial activity using the agar diffusion method [96,97] against representatives of Gram-positive bacteria (*Bacillus subtilis*) and Gram-negative bacteria (*Escherichia coli* and *Pseudomonas aeruginosa*) (Table 3). The test was performed using the diffusion agar technique—the well diameter was 6.0 mm (100 µL was tested), the positive control for bacteria was Gentamycin with a concentration of 4 µg/mL, and the sample concentration was 5 mg/mL.

From the data presented in Table 3, it is clear that all compounds under study have antibacterial activity upon Gram (+) and Gram (−) bacteria. As an example, compound **5** affects Gram-positive *B. subtilis* (zone of inhibition—19 mm) and Gram-negative *P. aeruginosa* (zone of inhibition—19 mm) weaker than Gram-negative *E. coli* (zone of inhibition—18 mm). In general, all of the synthesized compounds showed better antibacterial activity compared with the standard compound Gentamycin.

## 3. Materials and Methods

### 3.1. Materials

The starting reagents 1,8-naphthalic anhydride, 4-chloro-1,8-naphthalic anhydride, allyl amine, and hydrazine monohydrate were used as commercial products (Sigma-Aldrich Co., St. Louis, MO, USA and Fisher Scientific, Waltham, MA, USA) without purification. All used in the synthetic procedures and in the photophysical investigation solvents (Sigma-Aldrich Co., St. Louis, MO, USA and Fisher Scientific, Waltham, MA, USA) were pure or of spectroscopy grade.

### 3.2. Methods

Melting points were measured using an Electrothermal IA9100 (Profcontrol GmbH, Schönwalde-Glien, Germany) apparatus with open capillary tube and were uncorrected. The FT-IR spectra (KBr disk) were recorded on a Thermo Scientific Nicolet iS20 FTIR spectrophotometer (Thermo Fisher Scientific, Waltham, MA, USA). The ^1^H NMR and ^13^C NMR spectra were measured on a Bruker AV-600 spectrometer (BRUKER AVANCE II+ 600 MHz, Bruker, Billerica, MA, USA) with operating frequency at 600 MHz and 150.92 MHz using DMSO-*d*_6_ as a solvent. All chemical shifts were expressed on the *δ* (ppm) scale using TMS as an internal standard. The coupling constant (*J*) values are given in Hz. A Hewlett Packard 8452A spectrophotometer (Agilent Technologies, Inc., Santa Clara, CA, USA) was used for the UV-vis absorption measurements. The photophysical study was performed at room temperature (25.0 °C) in 1 × 1 cm quartz cuvettes. The fluorescence spectra were recorded using a Scinco FS-2 spectrofluorometer (Scinco, Seoul, Korea). The elemental analysis data were obtained on an automated EuroEA3000 CHNS-O Analyzer (Euro Vector S.P.A, Pavia PV, Italy). A very small volume of hydrochloric acid and sodium hydroxide was used to adjust the pH, which was monitored by HANNA instrument HI-2211 Bench Top pH meter (HANNA Instruments, Woonsocket, RI, USA).

### 3.3. Synthetic Procedures

#### 3.3.1. Synthesis of 4-Chloro-*N*-allyl-1,8-naphthalimide (**2**)

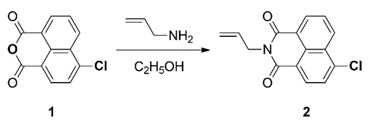



The 4-Chloro-1,8-naphthalic anhydride (**1**) (2.32 g, 0.01 mol) was dissolved in ethanol (50 mL) at 55 °C, and allyl amine (0.012 mol, 20% excess) was added. The solution was refluxed for 3 h, the liquor was then cooled, and the product that precipitated was filtered off, washed with water, and dried in vacuum at 30 °C to yield 4-chloro-*N*-allyl-1,8-naphthalimide **2** (2.55 g, 94%). FT-IR (KBr) cm^−1^: 3072 (νH-C=); 1699 (ν^as^N-C=O); 1662 (ν^s^N-C=O); 1588 (νC=C); 621 (νC-Cl). ^1^H NMR (DMSO-*d*_6_, 600.13 MHz) ppm: 8.48 (dd, 1H, *J* = 7.4 Hz, *J* = 1.1 Hz, naphthalimide H-5); 8.42 (dd, 1H, *J* = 8.2 Hz, *J* = 1.1 Hz, naphthalimide H-7); 8.20 (d, 1H, *J* = 7.9 Hz, naphthalimide H-2); 8.01 (dd, 1H, *J* = 8.2 Hz, *J* = 7.4 Hz, naphthalimide H-6); 7.79 (d, 1H, *J* = 7.9 Hz, naphthalimide H-3); 5.91 (m, 1H, allyl CH=); 5.08–5.03 (m, 2H, allyl CH_2_=); 4.68 (d, 2H, *J* = 4.9 Hz, allyl NCH_2_). Elemental analysis: calculated for C_15_H_10_ClNO_2_ (MW 271.70) C 66.31, H 3.71, N 5.16%; found C 66.19, H 3.66, N 5.23%.

#### 3.3.2. Synthesis of 4-Hydrazinyl-*N*-allyl-1,8-naphthalimide (**3**)

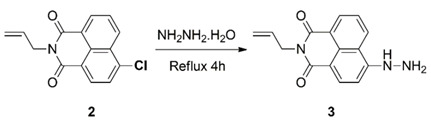



The 4-Chloro-*N*-allyl-1,8-naphthalimide **2** (0.82 g, 3.0 mmol) was added to 5 mL of hydrazine monohydrate. The reaction mixture was refluxed for 4 h under stirring and then poured into water. The precipitate was collected by filtration, washed with water, and dried to yield 4-hydrazinyl-*N*-allyl-1,8-naphthalimide **3** as a yellow solid (0.78 g, 97%). FT-IR (KBr) cm^−1^: 3433 and 3352 (νNH_2_); 3268 (νNH); 1673 (ν^as^N-C=O); 1633 (ν^s^N-C=O). ^1^H NMR (DMSO-*d*_6_, 600.13 MHz) ppm: 8.60 (dd, 1H, *J* = 8.1 Hz, *J* = 1.2 Hz, naphthalimide H-7); 8.42 (dd, 1H, *J* = 7.5 Hz, *J* = 1.2 Hz, naphthalimide H-5); 8.27 (d, 1H, NH); 8.17 (d, 1H, *J* = 8.0 Hz, naphthalimide H-2); 7.64 (m, 1H, naphthalimide H-6); 7.41 (d, 2H, NH_2_); 6.84 (d, 1H, *J* = 8.0 Hz, naphthalimide H-3); 5.92 (m, 1H, allyl CH=); 5.08 (m, 2H, allyl CH_2_=); 4.61 (d, 2H, *J* = 5.7 Hz, allyl NCH_2_). Elemental analysis: calculated for C_15_H_13_N_3_O_2_ (MW 267.28) C 67.40, H 4.90, N 15.72%; found C 67.65, H 4.86, N 15.59%.

#### 3.3.3. Synthesis of Dyad **5**

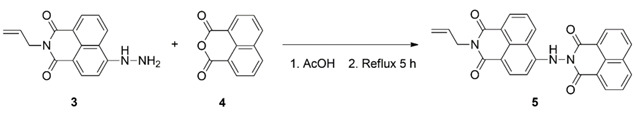



To a solution of 1,8-naphthalic anhydride **4** (0.51 g, 2.6 mmol) in 20 mL of acetic acid, 4-hydrazinyl-*N*-allyl-1,8-naphthalimide **3** (0.70 g, 2.6 mmol) was added. The resulting solution was refluxed for 5 h. After cooling, the yellow-orange precipitate was filtered off and dried to afford 1.02 g (88%) of probe **5**. FT-IR (KBr) cm^−1^: 3319 (νNH); 1702 and 1693 (v^as^N-C=O); 1662 and 1651 (v^s^N-C=O). ^1^H NMR (DMSO-*d*_6_, 600.13 MHz) ppm: 10.24 (br.s., 1H, NH); 8.81–8.57 (m, 4H, 2 × naphthalimide H-2, 2 × naphthalimide H-7); 8.55 (m, 2H, naphthalimide-2 H-4 and H-5); 8.20 (d, 1H, *J* = 8.4 Hz, naphthalimide-1 H-5); 8.11 (m, 2H, naphthalimide-2 H-3 and H-6); 7.89 (t, 1H, *J* = 7.9 Hz, naphthalimide-1 H-6); 6.96 (d, 1H, *J* = 8.3 Hz, naphthalimide-1 H-3); 5.91 (m, 1H, allyl CH=); 5.09 (m, 2H, allyl CH_2_=); 4.62 (d, 2H, *J* = 5.6 Hz, allyl NCH_2_). ^13^C NMR (DMSO-*d*_6_, 150.92 MHz) ppm: 163.32; 162.59; 162.11; 161.84; 148.75; 138.45; 133.28; 133.05; 132.57; 131.81; 131.10; 130.96; 128.91; 128.84; 128.73; 128.63; 127.99; 125.63; 122.90; 122.14; 121.61; 119.31; 116.03; 112.13; 105.90; 41.47. Elemental analysis: calculated for C_27_H_17_N_3_O_4_ (MW 447.44) C 72.48, H 3.83, N 9.39%; found C 72.24, H 3.79, N 9.41%.

### 3.4. Antibacterial Screening of the Synthesized Compounds

The biological activity of the resulting compounds was studied on Gram (+) (*Bacillus subtilis*) and Gram (−) *(Escherichia coli and Pseudomonas aeruginosa*) bacteria. The experiment was performed by using nutrient agar media (solid media prepared using yeast extract, agar, beef extract, sodium chloride, and peptone in distilled water) in different dishes—one for Gram (+) and the other for Gram (−). In the core of the media in every dish, a pore was made, and then 0.005 g/L (in DMF) of solid material was added to the pore. The dishes were put at 37 °C for 24 h, the inhibition zone was measured in every dish, and the results were tabulated to know the direct effect of the materials [96,97].

## 4. Conclusions

In conclusion, here was presented the synthesis of novel 4-iminoamido-1,8-naphthalimide fluorophore as an ICT-based colorimetric and fluorescence probe for the determination of pH in aqueous solutions. The target compound was designed on a “*fluorophore-receptor*” model where 4-imino-1,8-naphthalimide is the fluorophore and the iminoamido group, possessing a labile proton, is the receptor fragment. It can be summarized that at the transition from acidic to alkaline conditions, the absorbance of the probe was bathochromically shifted by 104 nm, and the color of the probe changed from yellow to red. Simultaneously, under these conditions, the probe fluorescence was quenched. The calculated fluorescence quenching of the probe was more than 60-fold. The analysis of the absorbance changes at 508 nm and fluorescence changes at 504 nm in a pH window 7–14 gave very similar p*K*_a_ values, which suggests a simple equilibrium by the deprotonation of the system. Moreover, based on the absorption and fluorescence behavior as a function of pH, the novel compound is able to operate as a two-input logic gate executing INHIBIT and IMPLICATION functions. In addition, all of the synthesized compounds showed better antibacterial activity toward Gram (+) and Gram (−) bacteria than the Gentamycin standard.

The conducted studies have indisputably demonstrated the high potential of the new compound as a reliable indicator for rapid detection of pH in aqueous solutions and base vapors in solid states.

## Figures and Tables

**Table 1:** Yields and characteristics of the synthesized compounds (**2**, **3**, and **5**) in water/DMF (3:1, *v*/*v*) solution.

Compound	Yield (%)	M.p. (°C)	*R*_f_	*λ*_A_ (nm)	*λ*_F_ (nm)
**2**	94	120–122	0.80 ^1^	342	-
**3**	97	228–230	0.70 ^2^	448	538
**5**	88	341–343	0.33 ^2^	342 404	410 504

^1^ TLC in the system acetone: *n*-heptane = ½; ^2^ TLC in the system acetone: *n*-heptane = 1/1.

**Table 2:** Truth table for logic gates of **5** with two chemical inputs (H^+^ and HO^−^).

Input 1 (H^+^)	Input 2 (HO^−^)	Output 1 (A_404_)	Output 2 (A_508_)	Output 3 (FI_504_)
0	0	0	1	0
1	0	1	0	1
0	1	0	1	0
1	1	0	1	0
		**INHIBIT**	**IMPLICATION**	**INHIBIT**

**Table 3:** Antibacterial activity of compounds **2**, **3**, and **5** against Gram (+) and Gram (−) bacteria. Mean zone of inhibition (IZ) in mm.

Compound	2	3	5	Control	
Tested microorganisms	Mean IZ	Mean IZ	Mean IZ	Mean IZ	±SD
Gram-positive bacteria:				Gentamycin	
*Bacillus subtilis:* RCMB 015 (1) NRRL B-543	14	15	19	26.02	0.03
Gram-negative bacteria:				Gentamycin	
*Escherichia coli*: (RCMB 010052) ATCC 25955	15	18	18	29.90	0.01
*Pseudomonas aeruginosa*	12	17	19	21.01	0.04

## Data Availability

The authors declare that the data supporting the findings of this study are available within the article.

## Supplementary Materials

The following supporting information can be downloaded at: https://www.mdpi.com/article/10.3390/molecules28083631/s1, Table S1: Photophysical characteristics of compound **5** in solvents of different polarity; Figure S1: Titration plot of dyad **5** at *λ*_A_ = 508 nm in a pH range ca. 2–14; Figure S2: Titration plot of dyad **5** at *λ*_F_ = 504 nm in a pH range ca. 2–14 (*λ*_EX_ = 400 nm); Figure S3: IR spectrum of compound **3**; Figure S4: ^1^H NMR spectrum of compound **3**; Figure S5: IR spectrum of dyad **5**; Figure S6: ^1^H NMR Spectrum of dyad **5**; Figure S7: ^13^C NMR Spectrum of dyad **5**; Figure S8: ^13^C NMR Spectrum of dyad **5** in a range of 164–138 ppm; Figure S9: ^13^C NMR Spectrum of dyad **5** in a range of 134–121 ppm; Figure S10 ^13^C NMR Spectrum of dyad **5** in a range of 122–105 ppm.
