# Supplementary material for: Design and Synthesis of a Novel ICT Bichromophoric pH Sensing System Based on 1,8-Naphthalimide Fluorophores as a Two-Input Logic Gate and Its Antibacterial Evaluation
Source: Molecules. 2023 Apr 21;28(8):3631. doi: 10.3390/molecules28083631 (PMC10145821; doi:10.3390/molecules28083631)
Supplement: Supplementary file 1 [file molecules-28-03631-s001.zip › molecules-2331767-supplementary.pdf]

SUPPLEMENTARY INFORMATION

Design and Synthesis of a Novel ICT Bichromophoric pH Sensing System Based on 1,8-Naphthalimide Fluorophores as a Two-Input Logic Gate and Its Antibacterial Evaluation

Table S1. Photophysical characteristics of compound **5** in solvents of different polarity.

	Chloroform				Acetonitrile			Ethanol			DMF	
**5**	λ_A (nm)	λ_F (nm)	Q_F		λ_A (nm)	λ_F (nm)		λ_A (nm)	λ_F (nm)		λ_A (nm)	λ_F (nm)
	340<br>378	360<br>496	0.35		342<br>390	375<br>498		342<br>402	380<br>500		342<br>404	395<br>516

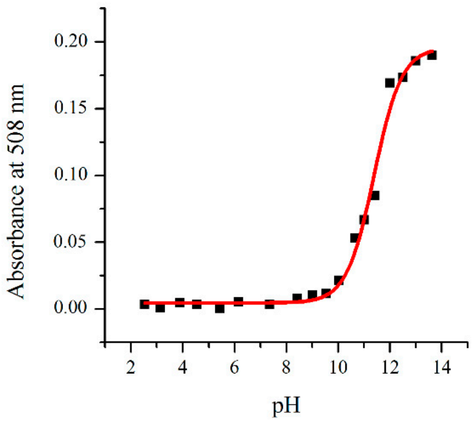

Figure S1. Titration plot of dyad **5** at λ_A = 508 nm in a pH range ca. 2-14.

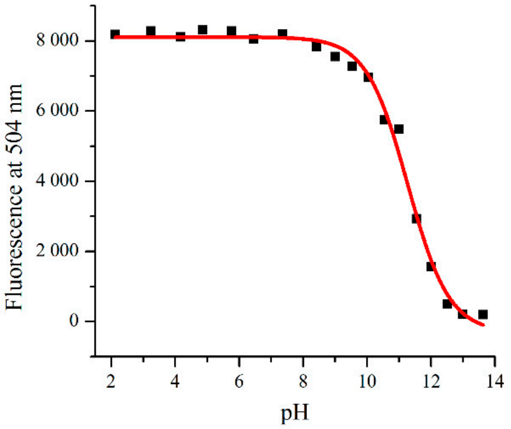

Figure S2. Titration plot of dyad **5** at λ_F = 504 nm in a pH range ca. 2-14 (λ_{EX} = 400 nm).

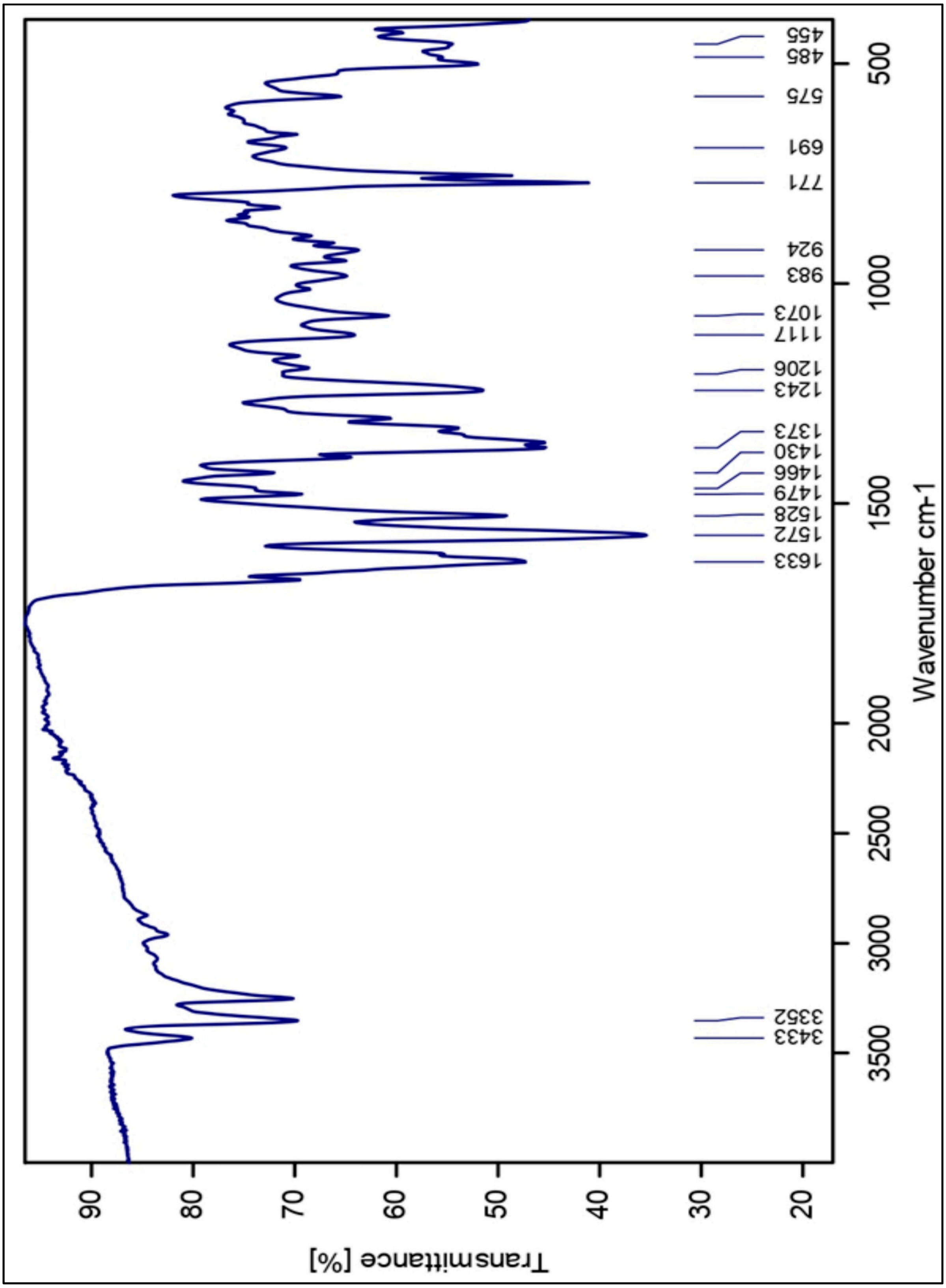

Figure S3: IR spectrum of compound **3**.

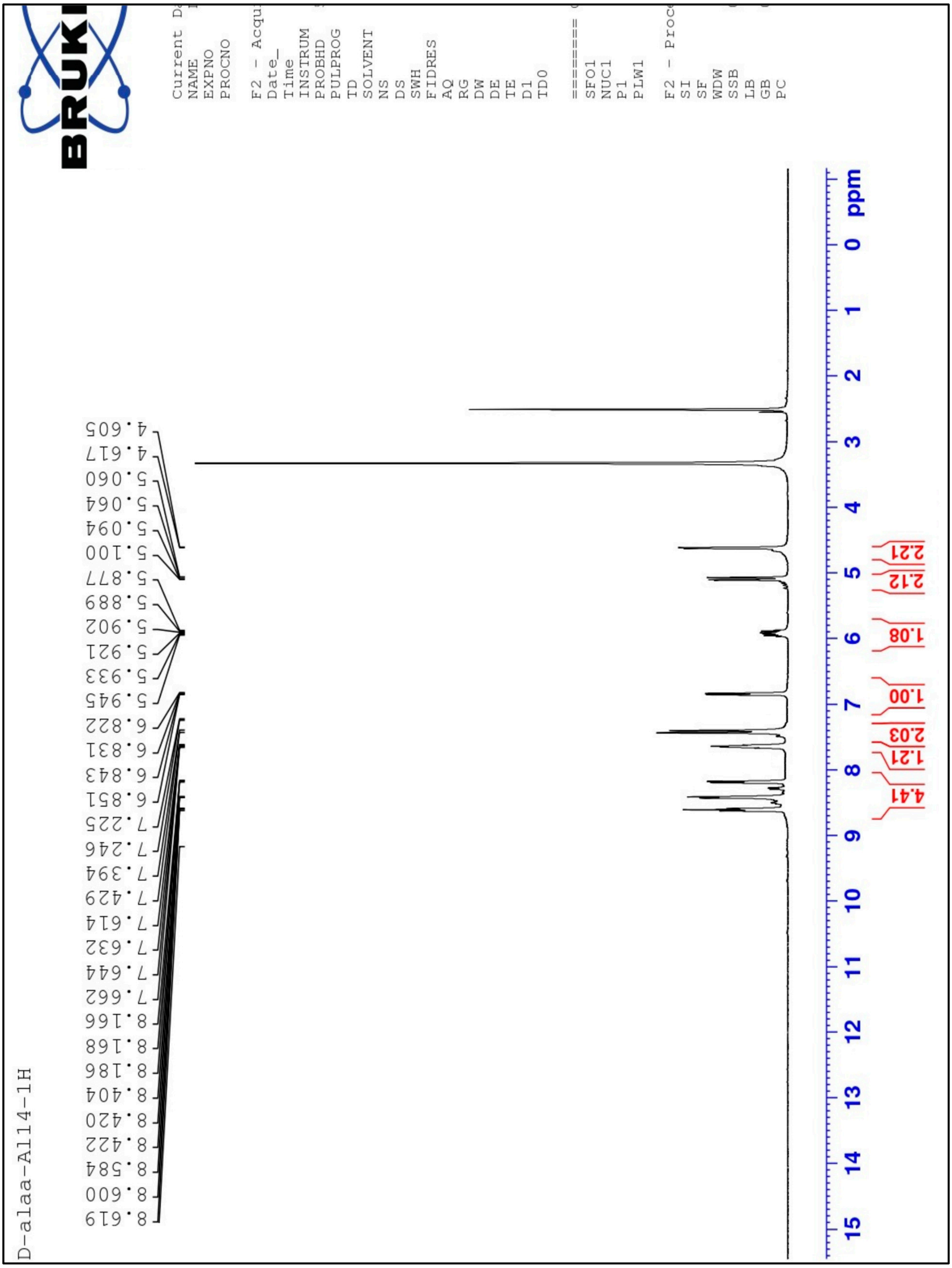

Figure S4. ^{1}H NMR Spectrum of compound **3**.

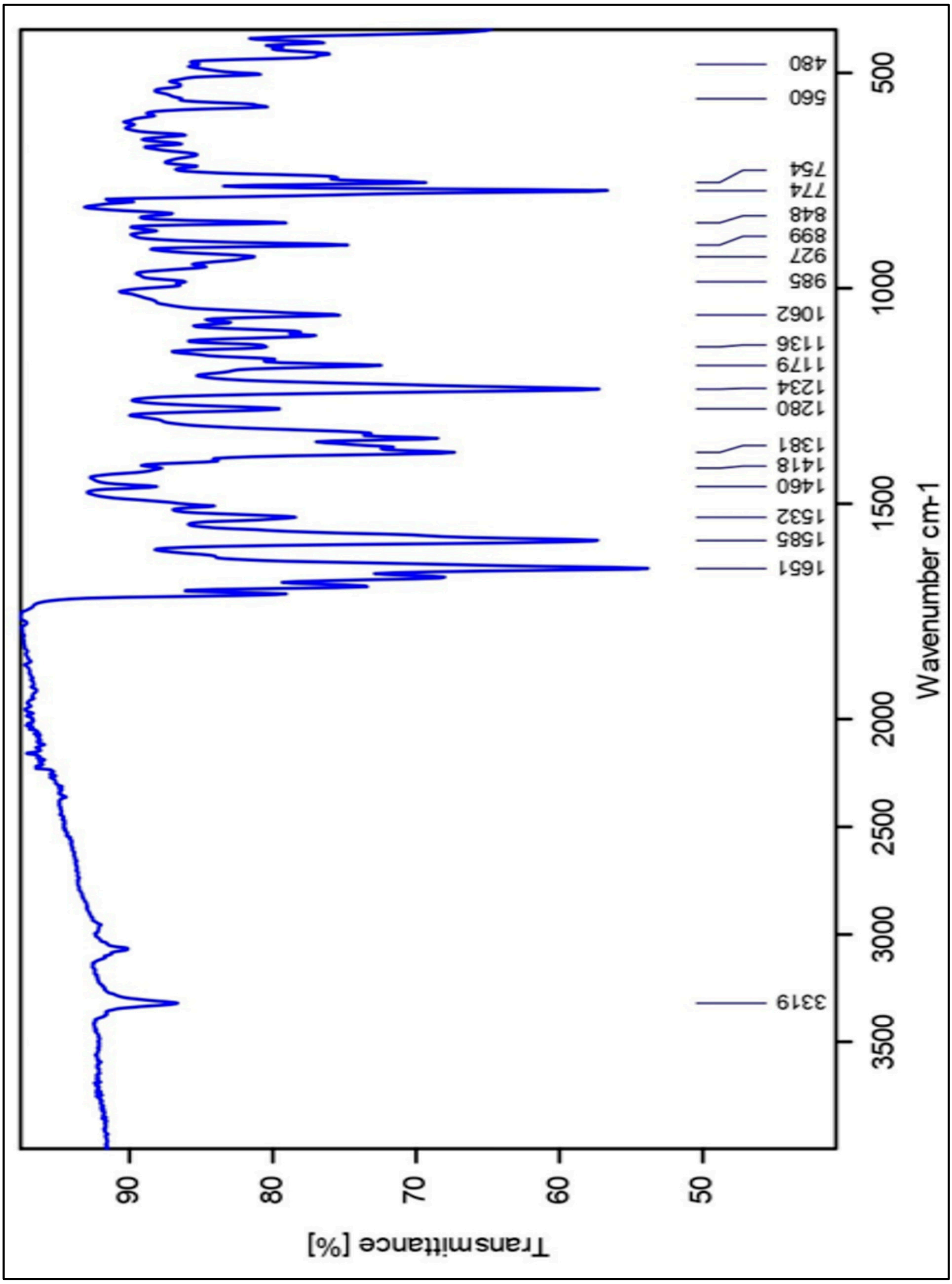

Figure S5: IR spectrum of dyad **5**.

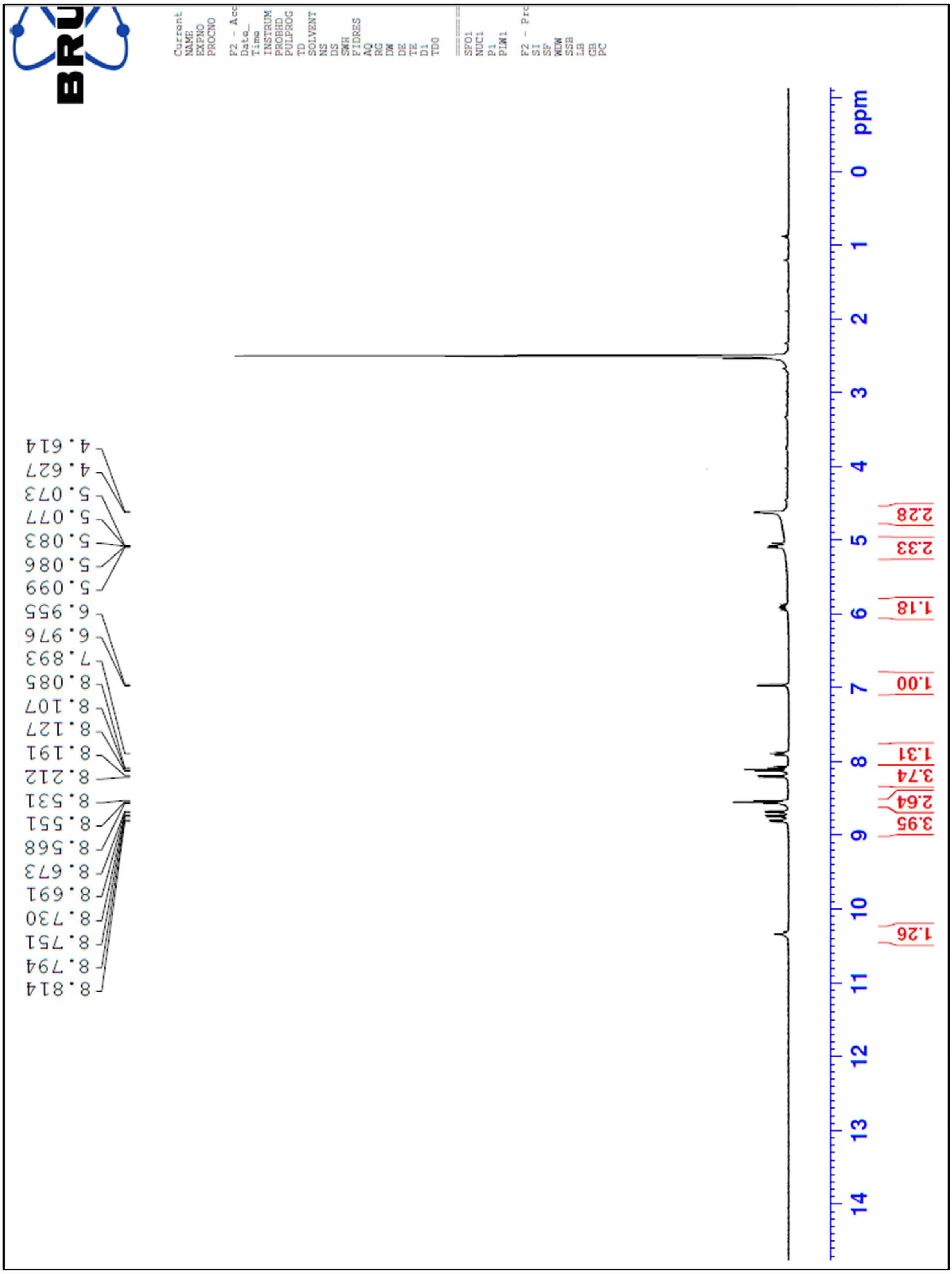

Figure S6. ^{1}H NMR Spectrum of dyad **5**.

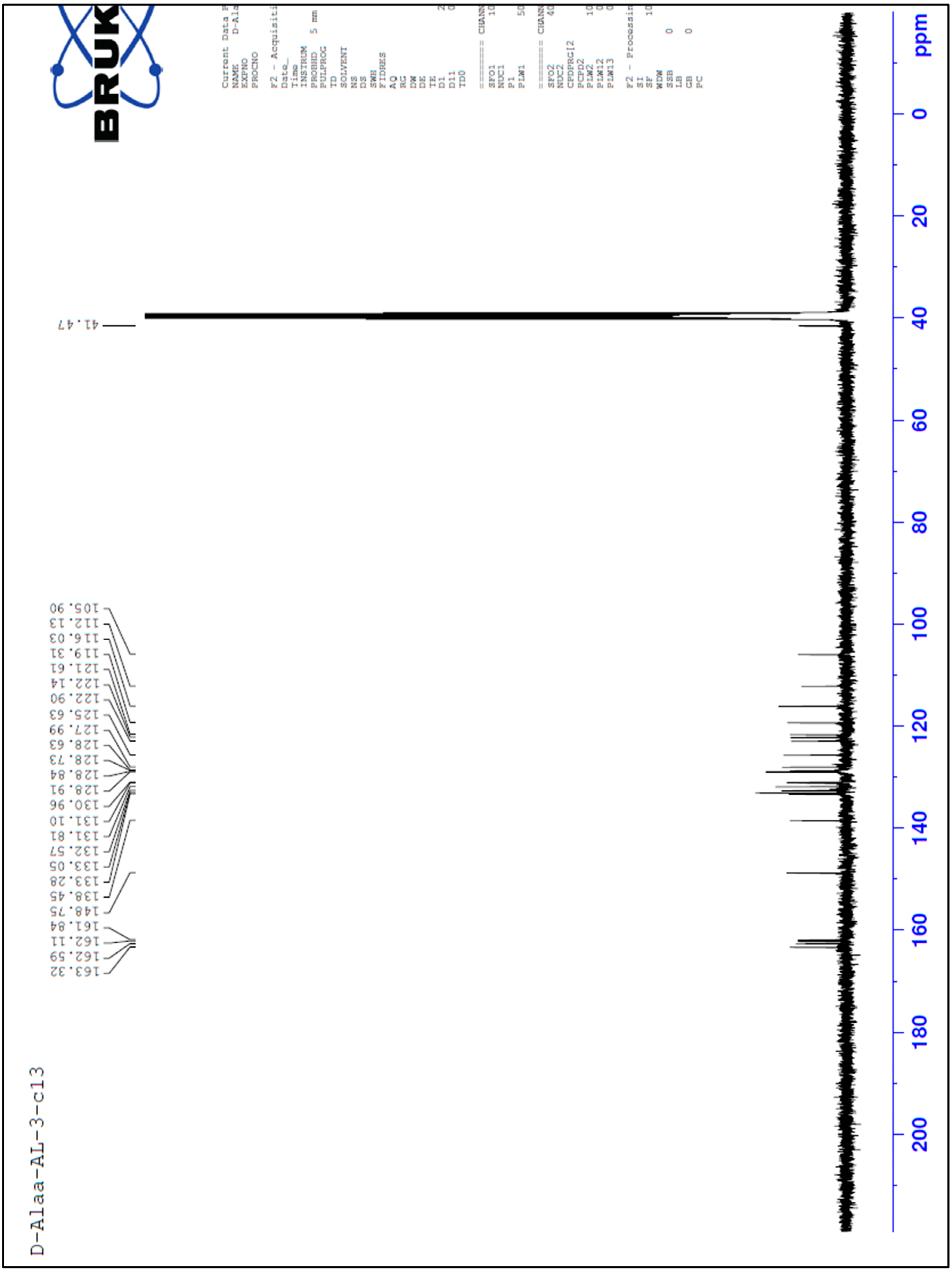

Figure S7. ^{13}C NMR Spectrum of dyad **5**.

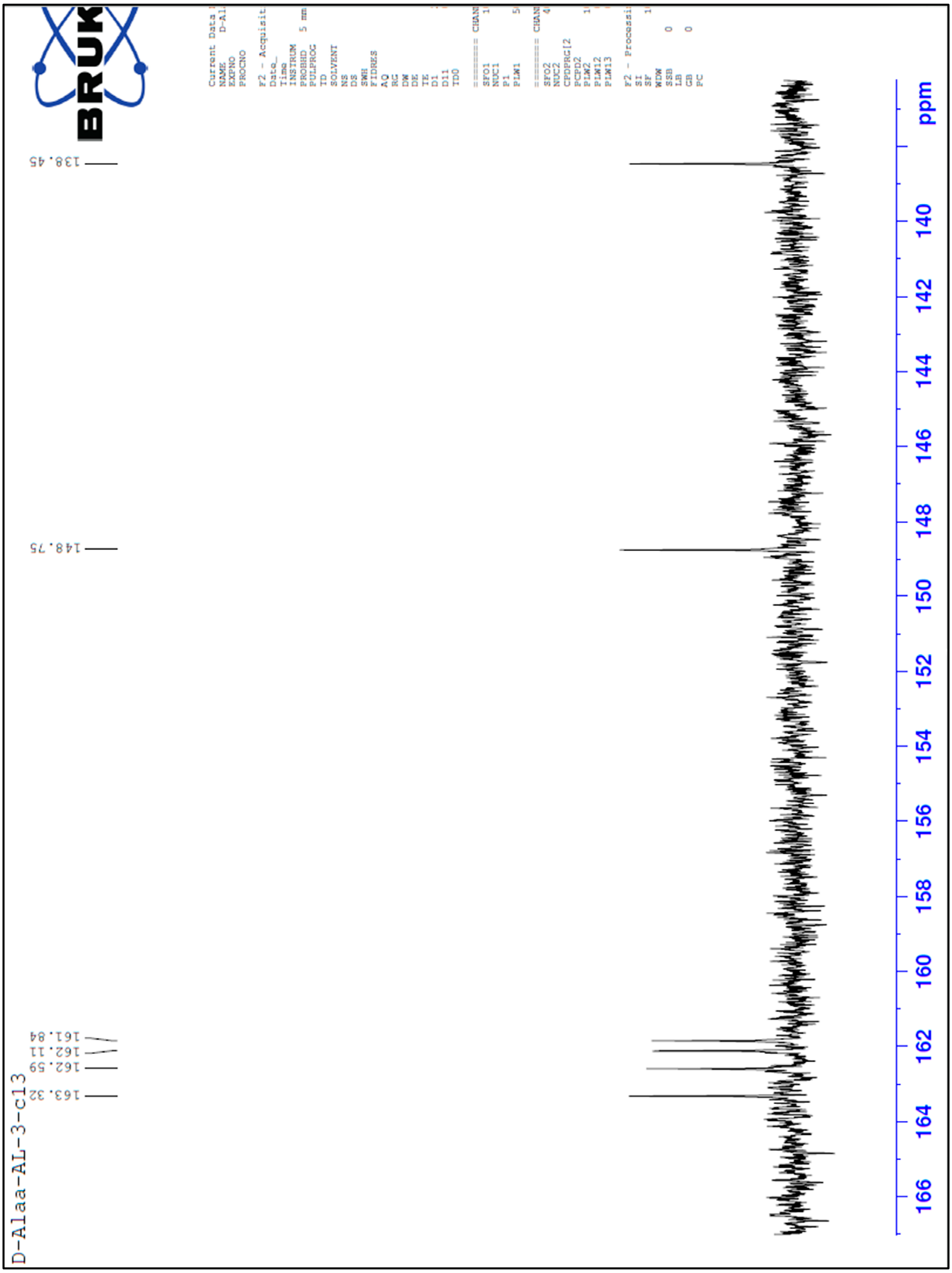

Figure S8. ^{13}C NMR Spectrum of dyad **5** in a range of 164-138 ppm.

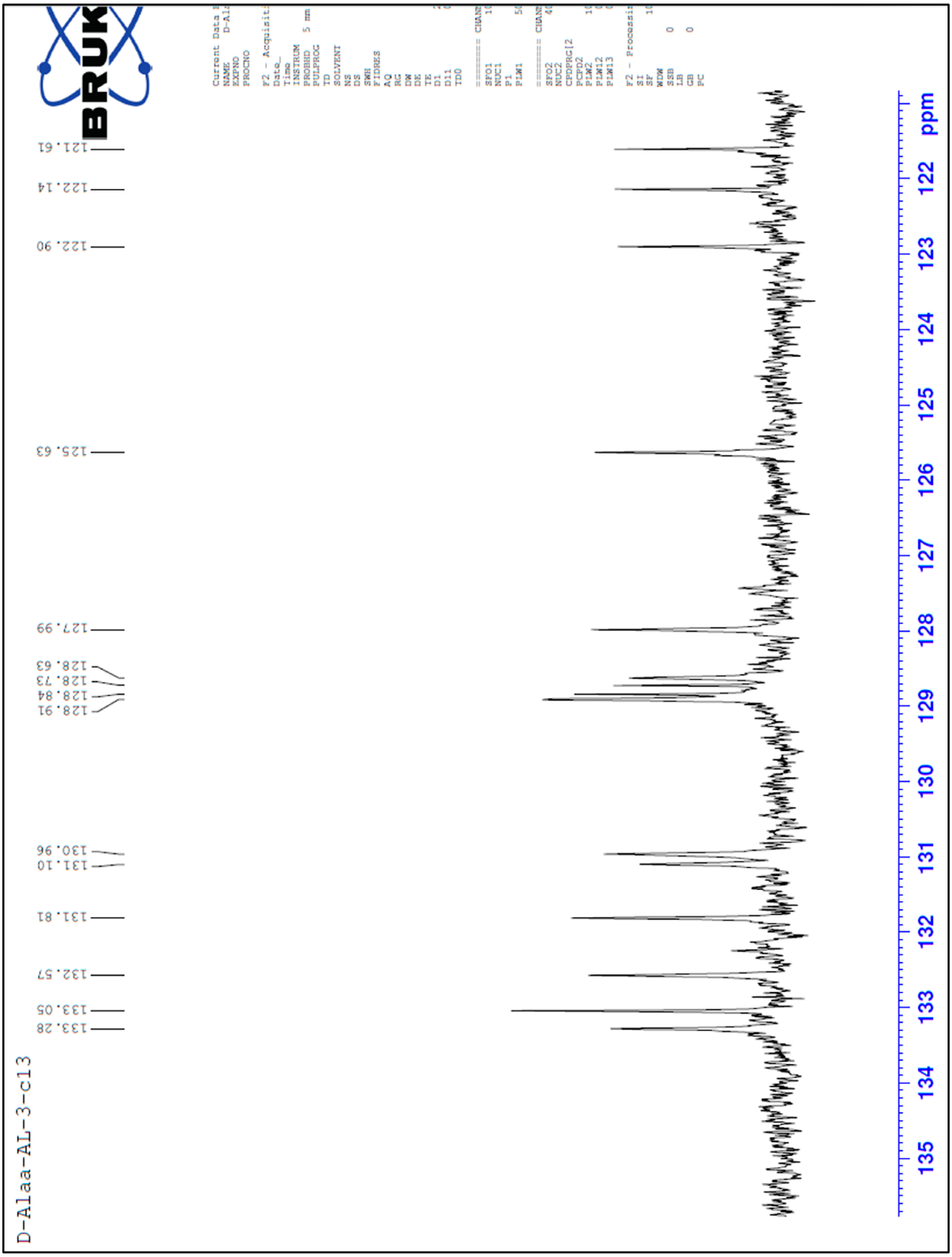

Figure S9. ^{13}C NMR Spectrum of dyad **5** in a range of 134-121 ppm.

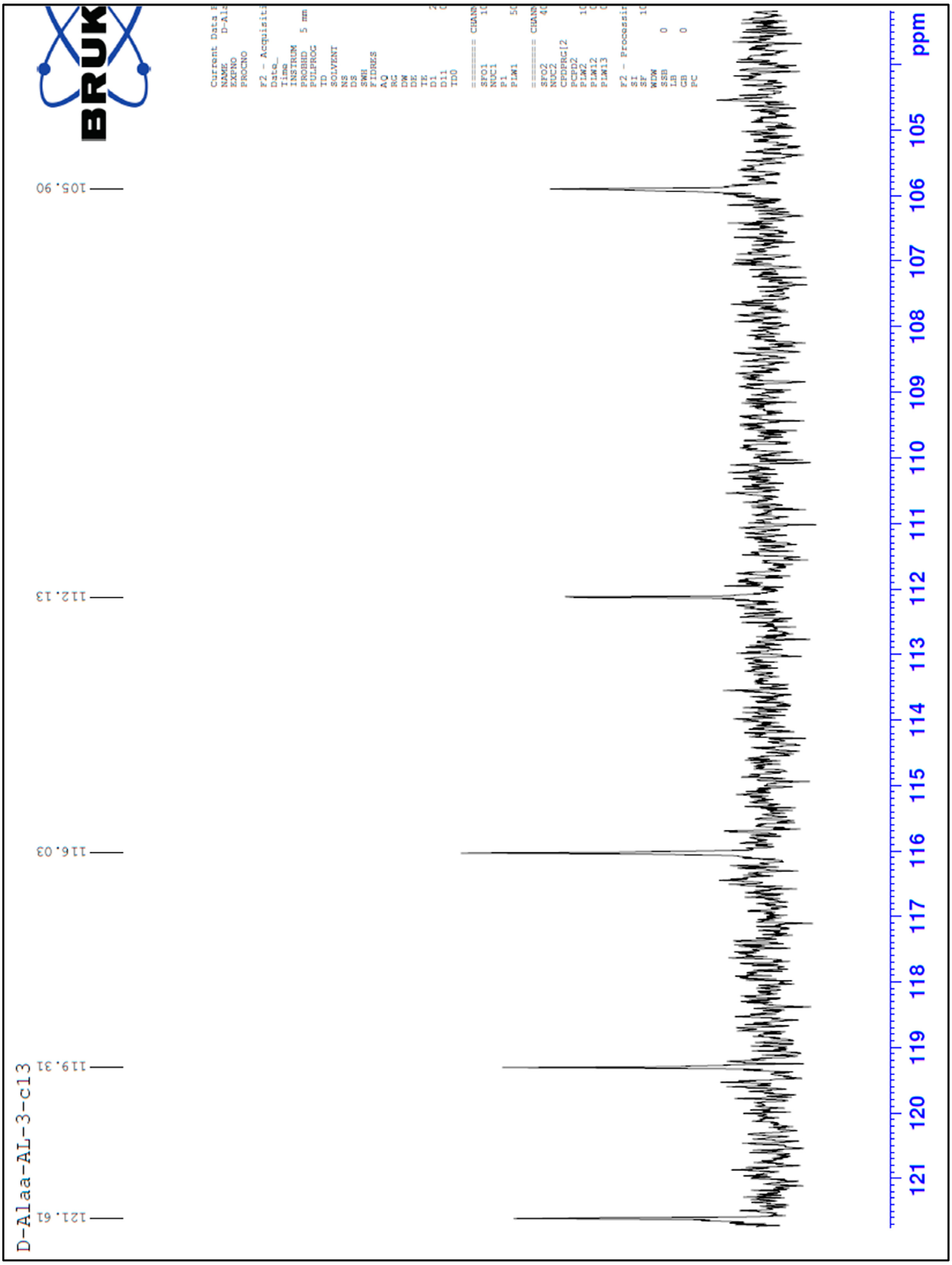

Figure S10. ^{13}C NMR Spectrum of dyad **5** in a range of 122-105 ppm.
